# Modeling Brain Tumors: A Perspective Overview of *in vivo* and Organoid Models

**DOI:** 10.3389/fnmol.2022.818696

**Published:** 2022-05-30

**Authors:** Francesco Antonica, Giuseppe Aiello, Alessia Soldano, Luana Abballe, Evelina Miele, Luca Tiberi

**Affiliations:** ^1^Armenise-Harvard Laboratory of Brain Disorders and Cancer, Department of Cellular, Computational and Integrative Biology (CIBIO), University of Trento, Trento, Italy; ^2^Laboratory of Translational Genomics, Department of Cellular, Computational and Integrative Biology (CIBIO), University of Trento, Trento, Italy; ^3^Department of Pediatric Hematology/Oncology and Cellular and Gene Therapy, Bambino Gesù Children’s Hospital, Scientific Institute for Research, Hospitalization and Healthcare (IRCCS), Rome, Italy

**Keywords:** organoid, mouse, *Drosophila*, xenograft, model, cancer, zebrafish

## Abstract

Brain tumors are a large and heterogeneous group of neoplasms that affect the central nervous system and include some of the deadliest cancers. Almost all the conventional and new treatments fail to hinder tumoral growth of the most malignant brain tumors. This is due to multiple factors, such as intra-tumor heterogeneity, the microenvironmental properties of the human brain, and the lack of reliable models to test new therapies. Therefore, creating faithful models for each tumor and discovering tailored treatments pose great challenges in the fight against brain cancer. Over the years, different types of models have been generated, and, in this review, we investigated the advantages and disadvantages of the models currently used.

## Introduction

**FIGURE 1:**
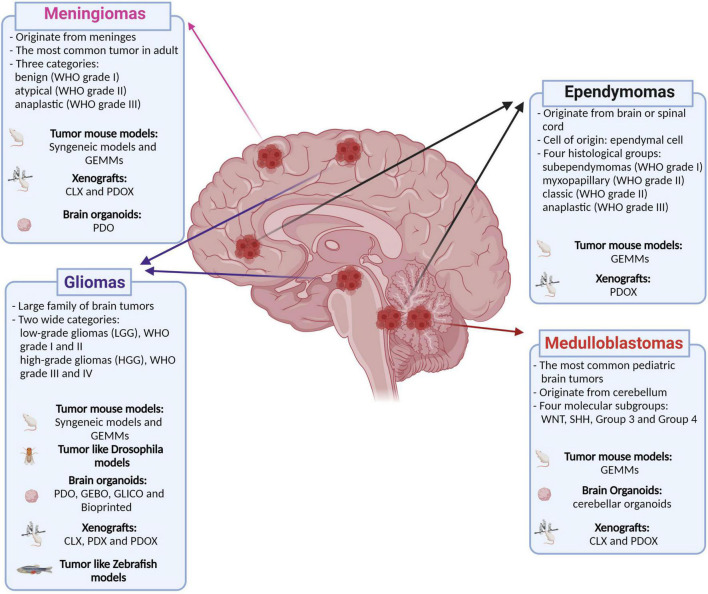
Classification of the main brain tumors discussed in this review and their relative available models. Genetically engineered mouse models (GEMMs); patient-derived organoids (PDO); genetically engineered brain organoids (GEBO); co-culturing GBM-derived GSCs with brain organoids (GLICO); cell lines xenografts (CLX); patient-derived xenografts (PDX); patient-derived orthotopic xenografts (PDOX). Created with BioRender.com.

Primary malignant brain tumors remain among the deadliest form of cancers despite the deeper understanding of their tumorigenic processes, acquired during the recent years, and the multimodality therapeutic approach (Aldape et al., 2019). Brain tumors are also the most common solid tumor in children and are the leading cause of cancer-related morbidity and mortality in this population. An important shared feature of this heterogeneous group of diseases is the unique biology of the brain and its microenvironment, which represents a further degree of complexity in understanding the underlying biological mechanisms and in generating and delivering effective therapies. To this end, genetically engineered mouse models (GEMM) have been widely used in the field of cancer research as they allow to study mechanisms of tumorigenesis and tumor biology in a physiological context (Li and Langhans, 2021). However, most of these models cannot fully recapitulate the human tumor heterogeneity and show great limitations for preclinical drug testing (Gould et al., 2015; Aldape et al., 2019). Patient-derived xenografts (PDX) have been generated to overcome these limitations and to resemble human cancer more closely. These models retain patient mutational heterogeneity and, to date, have been considered one of the most reliable models for preclinical tests (Brabetz et al., 2018; Smith et al., 2020). However, PDXs cannot be used to address mechanisms of tumorigenesis, as they are already derived from tumor tissue. Human-induced pluripotent stem cell (hiPSC)-derived organoids solve the latter limit and represent a great advance for the understanding of tumorigenesis and for the development of new therapeutic strategies (Bian et al., 2018; Ogawa et al., 2018; Ballabio et al., 2020). In this review, our aim is to describe the advantages and disadvantages of current and developing models for brain tumors with the highest incidence (meningiomas) or morbidity and mortality (gliomas, medulloblastoma, and ependymomas) in adulthood and childhood that could lead to new therapeutic strategies (Figure 1).

## Gliomas

Gliomas are a large family of brain tumors affecting adult as well as pediatric patients. Gliomas can be subdivided into two wide categories, low- and high-grade gliomas, based on their aggressiveness (Omuro and DeAngelis, 2013). The low-grade tumors (LGGs, Grades I and II according to the World Health Organization – WHO) are characterized by slower growth and infiltration compared with the high-grade (WHO Grades III and IV) (Louis et al., 2021). Nevertheless, the proper grade and the classification of the different gliomas rely not only on their histological features but also on the molecular characteristics such as genes found altered (i.e., *IDH1, ATRX, TP53, CDK2A, BRAF, FGFR1*, and *PDGFRA*) and on the methylation profile. In 2021, an updated edition of the WHO Classification of Tumors of the Central Nervous System was published that redefined all the pre-existing tumor classification (i.e., subclassification in adult vs. pediatric) and added new entities (i.e., diffuse glioma G34-mutant) (Louis et al., 2021). While LGGs show a better prognosis due to their low proliferation rate and infiltration, high-grade gliomas (HGGs) remain very challenging to cure with a median survival of less than 15 months in the case of the more aggressive glioblastoma multiforme (GBM), and diffuse midline glioma, H3 K27-altered in pediatrics. Indeed, the invasive nature of GBM makes surgical resection difficult; relapse occurs rapidly after treatments, and the high intra-tumor heterogeneity increases the difficulties in establishing effective drugs. The heterogeneity within GBM tumors has been identified at several levels, such as genetic, transcriptional, and DNA methylation. Since 2010, GBM has begun to be classified into 4 subtypes: proneural, neural, classical, and mesenchymal (Verhaak et al., 2010). This classification relied on peculiar aberrations and gene expression on specific genes, such as PDGFRA/IDH1, EGFR, and NF1. Afterwards, the improvement of high-throughput sequencing as well as the development of new profiling method (i.e., DNA methylation) has allowed to better stratify all the 4 canonical GBM subtypes (Zhang et al., 2020). In addition, it was shown how the prognosis can be closely correlated with the molecular subtype; for example, the proneural GBM has been shown to have a better prognosis than mesenchymal GBM (Phillips et al., 2006). The poorest prognosis of mesenchymal GBM is characterized by over-expression of genes related to angiogenesis and cell invasion (Phillips et al., 2006). Finally, using scRNA-seq technique has been defined that GBM has a peculiar feature of intra-tumoral heterogeneity. Indeed, several studies have shown that the different GBM molecular subtypes can co-exist within the same tumor (Patel et al., 2014; Darmanis et al., 2017; Neftel et al., 2019). This last aspect makes challenging to define which therapy would be more appropriate for each patient. Due to the inner complexity and aggressiveness, the development of reliable models for HGGs and GBM remains a crucial challenge in the tumor biology field, and several efforts have been conducted to generate *in vitro* and *in vivo* models, aiming to set up the best experimental strategy for investigating new therapeutic approaches (Ho et al., 2014; Hicks et al., 2021). On the other hand, low-grade gliomas, due to their low proliferative features, make challenging to culture them, as cell lines or organoids or to have a proper tumor growth in a reasonable period in a mouse model, and, for these reasons, are less investigated. Finally, the recent fine re-classification of the different glioma based on their genetic should be taken in consideration, for example, for the future creation of new genetic models.

### Mouse Models of Glioma

In the context of gliomas, different types of *in vivo* models have been generated until now: xenografts including CLX (cell lines xenografts) and PDX (patient-derived xenografts), syngeneic, GEMM, and *Drosophila melanogaster*.

#### Xenografts (Cell Lines Xenografts and Patient-Derived Xenografts)

**FIGURE 2:**
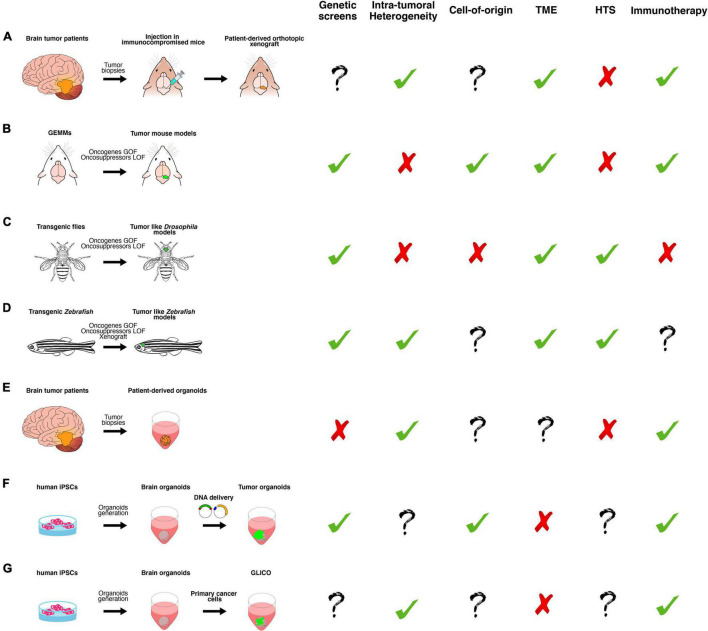
A schematic overview of the main classes of *in vitro* and *in vivo* preclinical brain cancer models and their relative applications. **(A)** Brain cancer tissue surgically resected from patients is directly transplanted in the brain of immunocompromised mice, producing patient-derived orthotopic xenograft models (PDOX). **(B)** Genetically engineered mouse models (GEMMs) in which the tumor formation is induced by gain or loss of function of oncogenes or oncosuppressors, respectively. They can be generated by breeding animals that carry germline mutations or injecting virus or plasmids, harboring the gene of interest. **(C)** Transgenic flies in which the tumor-like phenotype is determined by gain or loss of function of oncogenes or oncosuppressors, respectively, in a time- and tissue-specific manner. **(D)** Transgenic Zebrafish in which the tumor-like phenotype is determined by gain or loss of function of oncogenes or oncosuppressors, respectively, in a time- and tissue-specific manner. Xenotransplantation of human glioma cells in zebrafish. **(E)** Brain cancer tissue surgically resected from patients is directly cultured in 3D culture as patient-derived organoids (PDO). **(F)** Tumor organoids can be generated by gain or loss of function of oncogenes or oncosuppressors, respectively, in cerebral or cerebellar organoids derived from human-induced pluripotent stem cells (iPSCs). **(G)** GLICO can be generated by co-culturing primary cancer cells with cerebral organoids. Based on published works, we summarized the possible usages of each class of the models in the following applications: genetic screens (i.e., testing the function of new genes in tumor formation, progression and aggressiveness); investigation of intra-tumoral heterogeneity, cell of origin and tumor microenvironment (TME); high-throughput screening (HTS) of new chemotherapeutic drugs and, finally, for testing new immunotherapy approaches.

Xenograft models have been generated by injecting patient-derived cells or established cell lines into the mouse brain (i.e., U251, U87, A172, and T98G) (Table 1) (Fareh et al., 2012; Jandial et al., 2018; Hicks et al., 2021). Nevertheless, all the models based on cell lines lack the heterogeneity present in the human tumor due to the selection occurring in cells when cultured. A further step was taken when glioma stem-like cells (GSC) were isolated and maintained *in vitro* in 2D or in spheroid culture conditions. Indeed, CD133/PROMININ-1 positive cells can be isolated from primary tumors, cultured and propagated as spheres, and finally grafted in mice (Singh et al., 2004; Yi et al., 2016). Xenograft models can also be obtained by primary tumors (Sasaki et al., 2001; Joo et al., 2013; Kerstetter-Fogle et al., 2020) (Figure 2A and Table 1). These models, called PDX, have the enormous advantage of being directly generated from tumor cells, thus maintaining the features of the original neoplasm, including its cellular composition that will grow in the murine microenvironment (Shu et al., 2008). Although xenograft models are considered a reliable scenario and, in some cases, the closest to humans, they have the important weakness of being devoid of the host’s immune system, as they are generated in immune-compromised mice. More in detail, depending on the immunocompromised mouse strain used, the lack of adaptive or innate immune system does not allow investigating the interaction between tumor and immune system. A possible solution has been recently proposed by xenografting tumor cells into the telencephalon ventricle of wild-type mouse embryos, where tumors persist in postnatal life (Hoffmann et al., 2020). Furthermore, the growth of GBM cells in the brain of nude mice, coupled with the possibility of genetically labeling them with barcodes, has provided a novel approach to trace different tumor clones *in vivo*. Indeed, Lan et al. (2017) using the patient-derived orthotopic xenografts (PDOX)-based approach showed the contribution of the different cancer cells in tumor formation, aggression, and therapy response. The possibility to track patient-derived GBM cell behavior *in vivo* with the barcoding approach has suggested the existence of a proliferative hierarchy, contributing to the tumor re-growth with a slow-cycling cancer stem cell subpopulation at its apex (Lan et al., 2017). On the other hand, PDXs have been also shown to be a valid approach for *in vivo* biobanking of pediatric brain tumors. Indeed, the xenografted tumor tissue closely recapitulated the histology, genetics, and drug sensitivity of the original tumor (Brabetz et al., 2018; Smith et al., 2020). Finally, orthotopic xenografts represent a valuable system to test the tumorigenicity of glioma-related mutations. In particular, the introduction of H3.3-K27M mutation in neural stem cells (NSCs) makes such cells capable to induce tumors formation upon transplantation in the mouse brain (Haag et al., 2021). Furthermore, distinct mutations in H3.3 trigger a tumoral phenotype, depending whether the NSCs derived from forebrain or brainstem modeling the possible different origins of pediatric high-grade glioma (Bressan et al., 2021).

**TABLE 1:** An example of different types of brain tumor models and their relative features.

Glioma models			
Brain tumor	Type of model	Features	References
HGG	CLX	Glioma cell-lines T98 and U87 xenograft	Jandial et al., 2018
HGG	CLX	Glioma cell-lines TG1 xenograft	Fareh et al., 2012
HGG	CLX/syngeneic	Syngeneic glioma cell lines GL261 xenograft	Ausman et al., 1970
HGG	PDX/PDOX	Xenograft of patient-derived GBM cells/neurospheres	Sasaki et al., 2001; Joo et al., 2013; Kerstetter-Fogle et al., 2020
Pediatric tumors	PDOX	Xenograft of patient-derived tumor cells (from different type of pediatric glioma)	Brabetz et al., 2018; Smith et al., 2020
HGG	GEMM	*Pdgfb* OE, *Trp53^–/–^*	Hede et al., 2009
HGG	GEMM	*Pdgfb* OE, *Ink4a/Arf^–/–^, Trp53^–/–^, Pten^–/–^*	Hambardzumyan et al., 2009
HGG	GEMM	*K-Ras* OE, *Ink4a/Arf^–/–^*	Uhrbom et al., 2005
HGG	GEMM	*EGFRvIII* OE, *Ink4a/Arf^–/–^, Pten^–/–^*	Zhu et al., 2009
HGG	GEMM	*EGFRvIII* OE, *V12-Ras, Pten^–/–^*	Wei et al., 2006
HGG	GEMM	*Nf1*^+/−^, *Trp53*^+/−^, *Pten*^+/−^	Kwon et al., 2008
HGG	GEMM	*H-Ras-V12* OE, *Akt* OE, *Trp53*^+/−^	Marumoto et al., 2009
LGG	GEMM	*Nras*^*G*12*V*^ OE, *Idh1*^*R*132*H*^ *OE*, sh*Atrx*, sh*Trp53*	Núñez et al., 2019
HGG	GEBO	*EGFRvIII* OE, *EGFR* OE, *CDKN2A^–/–^/CDKN2B^–/–^* or *NF1^–/–^, TP53^–/–^, PTEN^–/–^*	Bian et al., 2018
HGG	GEBO	*H-Ras*^*G*12*V*^ OE, *TP53*^–/–^	Ogawa et al., 2018
GBM	PDO	Patient-derived organoid	Jacob et al., 2020
	***Drosophila***		
GBM	Transgenic	*dEGFR*λ, *dp110CAAX*, *dPTEN*, *dRas85D*^*N17*^, *dRas85D*^*V*12^, *dRaf^GOF^* OE, dPTEN dsRNA	Read et al., 2009, 2013
GBM	Transgenic	*dRaf*^GOF^*, hFGFR3-hTACC3, dEGFR*^ACT^*, dPI3K*^ACT^**	Chen et al., 2018
	**Zebrafish**		
GBM	Transgenic	*HRAS*^*G*12*V*^, *KRAS*^*G*12*V*^ *EGFRvIII*, *BRAF*^*V*600*E*^ OE	Mayrhofer et al., 2017
Glioma	Transgenic	*KRAS*^*G*12*V*^ OE	Ju et al., 2015
Glioma	Transgenic	*DAAkt1*, *DARac1* OE	Jung et al., 2013
HGG/MPNSTs	Knockout	*nf1a*^+/−^; *nf1b*^–/–^; *tp53*^*e7*/*e7*^	Shin et al., 2012
GBM	CLX	Glioblastoma cell line U373-MG with shRacs or Racs OE Xenograft	Lai et al., 2017
GBM	CLX	Glioblastoma cell line U87 and its derived cancer stem cells Xenograft	Yang et al., 2013a
GBM	CLX	Glioblastoma cell lines U87 and U373 Xenotransplantation alone or with Mesenchymal stem cells	Breznik et al., 2017
GBM	PDX	Primary GBM-derived cells xenografts	Rampazzo et al., 2013
GBM	PDX	glioblastoma GBM9 cells xenografts into *prkdc*^–/–^, *il2rga*^–/–^	Yan et al., 2019

**Medulloblastoma models**			

**MB subgroup**	**Type of model**	**Features**	**References**

WNT	GEMM	*Ctnnb1*^+/−^; *TP53*^–/–^	Gibson et al., 2010
WNT	PDOX	Xenograft of patient-derived tumor cells	Brabetz et al., 2018; Smith et al., 2020
SHH	GEMM	*Ptch1* ^+/−^	Goodrich et al., 1997
SHH	GEMM	P*tch1*^ + ⁣/−^, *Trp53*^–/–^	Wetmore et al., 2001
SHH	GEMM	*Ptch1*^+/−^, *Cdkn2c^–/–^*	Uziel et al., 2005
SHH	GEMM	*SmoA2* OE	Dey et al., 2012
SHH	GEMM	*Sufu*^+/−^, *Trp53^–/–^*	Lee et al., 2007
SHH	GEMM	*Trp53*^–/–^ with *gPtch1.1*/Cas9	Zuckermann et al., 2015
Adult SHH	GEMM	*SmoM2* OE, *truncated BRPF1* OE	Aiello et al., 2019
SHH	Orthotopic xenograft	*MYCN* OE in NES	Huang et al., 2019
SHH	Orthotopic xenograft	Orthotropic xenograft of iPSC-derived NES from a Gorlin patient with a germline *PTCH1* mutation	Susanto et al., 2020
SHH	PDOX	Xenograft of patient-derived tumor cells	Brabetz et al., 2018; Smith et al., 2020
Group 3	GEMM	*Mycn* OE, *Trp53*^–/–^	Swartling et al., 2010
Group 3	GEMM	GTML *Trp53*^*KI*/*KI*^ *p53ER^TAM^*	Hill et al., 2015
Group 3	GEMM	*Mll4* ^–/–^	Dhar et al., 2018
Group 3	GEMM	*Gfi1* OE + *c-MYC* OE*/Otx2* OE + *c-MYC* OE	Ballabio et al., 2020
Group 3	Orthotopic xenograft	*Myc* OE, *Trp53*^–/–^	Kawauchi et al., 2012; Pei et al., 2012
Group 3	Orthotopic xenograft	*Myc*^*T*58*A*^ OE, *Trp53* DN	Pei et al., 2012

**Glioma models**			

**Brain tumor**	**Type of model**	**Features**	**References**

Group 3	Orthotopic xenograft	*Myc* OE, *GFI1b* OE	Northcott et al., 2014
Group 3	PDOX	Xenograft of patient-derived tumor cells	Brabetz et al., 2018; Smith et al., 2020
Group 3	Organoid model	*Gfi1* OE + *c-MYC* OE*/Otx2* OE + *c-MYC* OE	Ballabio et al., 2020, 2021
Group 4	GEMM	Activated SRC OE, *Tp53* DN	Forget et al., 2018
Group 4	PDOX	Xenograft of patient-derived tumor cells	Brabetz et al., 2018; Smith et al., 2020

**Ependymoma models**			

**Brain tumor**	**Type of model**	**Features**	**References**

Ependymoma	GEMM	*RELAFUS1*	Ozawa et al., 2018
Ependymoma	GEMM	YAP1-MAMLD1	Pajtler et al., 2019
Ependymoma	GEMM	*nlsYAP5SA* or *Lats1*^–/–^ and *Lats2*^–/–^	Eder et al., 2020
Ependymoma	PDOX	Xenograft of patient-derived tumor cells	Brabetz et al., 2018; Smith et al., 2020

**Meningioma models**			

**Brain tumor**	**Type of model**	**Features**	**References**

Meningioma	CLX	BenMen1, Me3TSC cell line xenograft	Püttmann et al., 2005; Cargioli et al., 2007
Meningioma	CLX	CH-157-MN cell line xenograft	Ragel et al., 2008
Meningioma	CLX	IOMM-Lee cell line xenograft	McCutcheon et al., 2000
Meningioma	PDOX	Xenograft of patient-derived tumor cells	McCutcheon et al., 2000
Meningioma	GEMM	*Nf2* ^–/–^	Kalamarides et al., 2002
Meningioma	GEMM	*PDGFB* OE + *Nf2^–/–^* + *Cdkn2ab*^–/–^	Peyre et al., 2015
Meningioma	GEMM	*SmoM2* OE	Boetto et al., 2018
Meningioma	GEMM	*Nf2^–/–^* + *Ink4a*^–/–^	Kalamarides et al., 2011
Meningioma	PDO	Patient-derived organoids	Chan et al., 2021; Yamazaki et al., 2021

#### Syngeneic Models

Several years ago, an interesting approach of inducing brain cancer in mice was developed, which consists of injecting ethyl-nitrosourea into the placenta of pregnant females between E15 and E18 (Thomas et al., 1994), or injecting methylcholantrene directly in the brain (Seligman et al., 1939). Both treatments with carcinogens lead to the formation of GBM-like tumors, from which cell lines are derived and, in turn, used to generate syngeneic allograft serially grafting (Ausman et al., 1970; Kaye et al., 1986). These models, contrary to PDOX where immunocompromised mice are used, allow to study the interaction between tumor-immune microenvironment and give the possibility to test immunotherapies. On the other hand, these models do not recapitulate several aspects of HGGs and GBMs, such as infiltration and histology. Recently, injection of engineered NSCs (*Nf1* and *Pten* KO + *EGFRvIII* overexpression) into immunocompetent syngeneic mouse strain has been used to propose an epigenetic-driven mechanism exploited by GBM stem cells to evade immune system (Gangoso et al., 2021).

#### Genetically Engineered Mouse Model

The genetically engineered mouse model (GEMM) represents one of the most reliable *in vivo* models to study whether specific genetic alterations are responsible for tumor initiation and progression (Figure 2B). The analysis of the genetic landscape of HGG and GBM led to the identification of the most frequently mutated or amplified genes, and specific GEMMs have been created based on this knowledge. Such genetic alterations include gene amplification (i.e., *PDGFA*/*B*), gain-of-function activating mutations (i.e., *KRAS*, *HRAS*, *EGFR*, *NRAS*, and *PDGFRA*) and loss-of-function mutations/gene deletions (i.e., *TP53*, *CDKN2A*, *PTEN*, *NF1*, *ATRX*, *AKT*, *IDH1*, *H3F3A*, and *INK4a*) (Table 1) (Uhrbom et al., 2005; Wei et al., 2006; Kwon et al., 2008; Hambardzumyan et al., 2009; Hede et al., 2009; Marumoto et al., 2009; Zhu et al., 2009; Núñez et al., 2019; Akter et al., 2021; Hicks et al., 2021). However, GEMMs also have several limitations. They are time-consuming and tumors do not always recapitulate the intra-tumor heterogeneity observed in patients (Reilly, 2009; Day et al., 2015).

##### Cre-Lox

Cre-lox is a gene-editing technology that allows site-specific recombination between sequences called Lox sites using the enzyme Cre recombinase. The action of Cre can be spatio-temporally controlled by driving its expression in certain cell types *via* specific promoters or Tamoxifen administration (when Cre is fused to *ER*^*T*2^). For example, this system has been used to generate mice that develop tumors by the introduction of the *EGFRvIII* genetic variant (Zhu et al., 2009), shedding light on the impact on GBM tumorigenesis of wild type and mutant forms of EGFR. The genetic knocking out of *Nf1, Trp53*, and *Pten* is also able to induce glioma in the mouse brain. Furthermore, the Cre-Lox approach helps to investigate the putative cell of origin by selective gene loss in specific lineage using peculiar promoters (Llaguno et al., 2009, 2015, 2019). The penetrance of brain tumor formation changes with the differentiation state of the cell, as observed in transgenic mice where tamoxifen-inducible Cre was expressed in neural stem cells (i.e., *Nestin-CreER*^T2^) (Llaguno et al., 2009), bipotential progenitors (i.e., *Ascl1-CreER*TM), oligodendrocyte progenitors (i.e., *NG2-creER*TM) (Llaguno et al., 2015) and immature (*NeuroD1-creER^T2^*) or post-mitotic neurons (*CamKII*α*-creER*TM) (Llaguno et al., 2019). Indeed, only neuronal stem cells (Nestin^+^ or GFAP^+^ cells) and early progenitors (Ascl1^+^ or NG2^+^ cells) that are at the top of the differentiation hierarchy are susceptible to cancer formation, while tumorigenesis is abolished in immature or post-mitotic neurons (NeuroD1^+^ or CamKIIα^+^ cells). Such an experimental model can be easily applied to the study of other tumors such as medulloblastoma and organoid models (described in the next sections).

##### Transposon System

Another method to generate GEMMs that overexpress or inactivate/silence the genes responsible for gliomagenesis is to insert the transgenes of interest into the genome, using the transposon system. The sleeping beauty (SB) transposon-based glioma mouse model was created, overexpressing *PDGF-A* and silencing *Nf1* and *Trp53* (Sumiyoshi et al., 2018). The same approach was used to test the role of *Atrx* silencing in brain tumors induced by overexpression of *Nras* and *Trp53* silencing (Koschmann et al., 2016). The possibility to integrate different constructs and deliver the DNA in the newborn mouse brain offers the advantage to test the tumor-inducing function of several genes in a less costly and time-consuming way.

##### RCAS-TVA

One of the most popular systems to generate GEMM to induce brain tumors is the RCAS-TVA method. RCAS-TVA is an efficient viral gene delivery system, consisting of RCAS (replication-competent avian sarcoma) viruses that carry the genes of interest and can only infect cells expressing the receptor TVA (tumor virus A). These cells were, indeed, previously engineered to express the tTA receptor under the control of specific cell lineage promoters, such as *Nestin*, *Gfap* (Holland et al., 1998), and *CNPase* (Lindberg et al., 2009). This allows studying whether specific oncogenes can induce tumor in a defined population and different anatomical regions. Of note, the RCAS-TVA approach has revealed how GBM can be induced by the *EGFR* mutant with loss of *Ink4a* or *Trp53* and that they arise more easily from *Nestin* cells than *Gfap* ones (Lindberg et al., 2009). The same approach has been used to model the low-grade brain tumor pilocytic astrocytoma (Gronych et al., 2011).

Globally, the GEMMs have the advantage to model GBM and HGG in mice with an intact immune system, allowing to test immunotherapies and to investigate the interaction between neoplastic and immune cells. Furthermore, GEMMs allow studying the impact of the different mutations on tumor progression and response to treatments. On the other hand, GEMMs have also serious and important pitfalls such as the lack of intra-tumor heterogeneity and the diversity of blood–brain barrier (BBB) in the mouse brain compared to the human one (Aday et al., 2016), thus affecting the treatment delivery.

### Drosophila melanogaster

*Drosophila melanogaster* is a powerful genetic model that can be successfully used to study cancer biology (Figure 2C and Table 1). Its intrinsic peculiarity allows overcoming the downsides of other models, such as tissue cultures and mice. In fact, the signalings involved in cancer are conserved in *Drosophila* (Reiter et al., 2001; Yamamoto et al., 2014), and a wide range of genetic tools exists to perform large-scale genetic screens. *Drosophila* represents also a powerful platform to perform pharmacological screens; in fact, compared to cell or organoid cultures, it represents a whole animal system that allows testing of a large number of compounds simultaneously in a high throughput fashion (Bell et al., 2009; Willoughby et al., 2013). Compared to the different techniques used to generate mouse models of brain tumors, fly gliomas have been studied by producing genetically modified *Drosophila* strains that carry alterations in the key pathways affected in human patients.

#### *Drosophila* Model of Glioma Obtained by Perturbation of EGFR-PI3K Signaling: A Tool to Understand Gliomagenesis

Many of the key signalings perturbed in GBM, such as EGFR signaling and the phosphatidyl-inositol-3-kinase (PI3K) pathway (Brennan et al., 2013), are conserved in *Drosophila.* Moreover, single functional orthologs exist for most of these genes, such as *EGFR* (*dEGFR*), *PIK3CA* (*dp110*), *PTEN* (*dPTEN*), *RAS* (*dRas*), *RAF* (*dRaf*), and *AKT* (*dAkt*). Glia-specific co-activation of EGFR and PI3K signaling in the fruit fly results in diffuse glial neoplasia in the larva (Read et al., 2009, 2013). This model has been used to perform a kinome-wide genetic screen that identified new genes involved in glioma development (Read et al., 2013). Interestingly, most of the modifiers have orthologs previously connected to GBM, while 16 appeared to be new modifiers, such as *dRIOK1* and *dRIOK2*. Overexpression of mutant *dEGFR* and *dp110* in precise time windows during adult life (Chi et al., 2018) also leads to brain enlargement and extensive glial expansion that ultimately results in a shorter lifespan and defective neural behaviors. Chen et al. (2018) established both high- and low-grade glioma models in the fruit fly by expressing constitutively active *Drosophila* Raf (dRaf^GOF^), human *FGFR3-TACC3* fusion gene, or constitutively active *Drosophila* EGFR and PI3K (dEGFR^ACT^; dPI3K^ACT^) in glia. Interestingly, the gliomas exhibited an increase of tissue stiffness compared with non-transformed brains, similar to the gradual tissue stiffening observed in human LGGs compared to HGGs (Miroshnikova et al., 2016) mediated by the Ion Channel dPiezo (Chen et al., 2018). The importance of tissue stiffness in glioma has also been demonstrated by Kim et al. (2014); through a genetic screening, they identified Lox, a Lysyl oxidase involved in extracellular matrix stiffness, as a potential mediator of neoplastic glial migration dependent on the pan-glial PDGF receptor (Pvr).

#### *Drosophila* Model of Glioma Obtained by Perturbation of EGFR-PI3K Signaling: A Tool to Understand the Cancer Stem Cells Role in Tumorigenesis

*Drosophila* has largely contributed to the understanding of the molecular mechanism underlying asymmetric cell division, an intrinsic property of cancer stem cells, leading to the first discoveries connecting this process to tumorigenesis (Caussinus and Gonzalez, 2005; Gómez-López et al., 2013). The previously described *Drosophila* GBM models driven by RTK-Ras have been used to identify and study transcription factors altered in GSCs. For example, Cheng et al. (2016) described the role of the transcription factor FOXD1 and of its target ALDH14A3, whose expression is altered in patient-derived GSCs. Another example of a transcription factor whose expression is altered in patient-derived GSCs is the Achaete-scute homolog 1 (ASCL1), an ortholog of the *Drosophila* achaete. Using the *Drosophila* GBM model described before (Read et al., 2009), it has been demonstrated that overexpression of fly Achaete or human *ASCL1* reduced tumor size and proliferation, and induced a switch from glial to neuronal fate (Park et al., 2017). Interestingly, the ASCL1 role is evolutionarily conserved; in fact, ASCL1 overexpression efficiently reduces growth capacity of proneural human cancer stem cells-derived models of GBM and promotes a lineage switch, activating the neuronal fate and repressing the glial one (Narayanan et al., 2019; Azzarelli et al., 2022).

Therefore, Park et al. (2017) showed the usefulness of *Drosophila* GBM models for understanding how transcription factors involved in differentiation processes can affect GBM formation and gliomagenesis. YAP (yes-associated protein, also known as YAP1) and TAZ (transcriptional co-activator with PDZ-binding motif) are Hippo pathway effectors involved in the control of stem cells fate (proliferation/differentiation). Using the previously described Pten-RNAi/RASV12 overexpression-induced GBM model (Cheng et al., 2016), Minata et al. (2019) demonstrated that Tep1 (CD109 in mammals) loss in glioma cells reduces Yki (the *Drosophila* YAP/TAZ ortholog) and attenuates gliomagenesis. High levels of CD109 have been reported in GBM samples, and this work clarified its role in clonogenicity, tumor initiation, and radio-resistance of GBM. The data obtained both in human and *Drosophila* samples (Gangwani et al., 2020) suggest a conserved oncogenic signaling of CD109 through the YAP/TAZ pathway. Understanding the role of this signaling in *Drosophila* is extremely relevant to human pathology as YAP/TAZ have been shown to be required for GSCs plasticity and for GBM initiation due to their ability to prevent GSC differentiation. Finally, Hakes and Brand (2020) clarified the contribution of neural lineages to GBM by studying the orphan nuclear receptor TLX, which correlates with a poor prognosis in patients with GBM (Park et al., 2010). TLX is required for a linage-specific (Type II) NSCs identity and progression during development and, when overexpressed, can induce tumor formation by inducing a switch in NSC identity, a block of differentiation and reversion of intermediate neural progenitors (INP)s to NSC fate.

In conclusion, *Drosophila* has been proved very helpful in studying the signaling pathway, and the molecular mechanisms underlying GBM development and the finding obtained using this model are of great relevance for the understanding of human pathogenesis. However, *Drosophila* still maintains many limitations in mimicking the human microenvironment. Indeed, the absence of an adaptive immune system as well as the differences in the ability of the immune cells to infiltrate the brain tissues limits the possibility to study the immune-tumor interaction. Moreover, due to the differences between humans and *Drosophila* development, this model does not allow to fully recapitulate human brain tumor heterogeneity and pathology.

### Zebrafish (*Danio rerio*)

Zebrafish is a well-established and robust model for studying cancer pathology and its fit for rapid and efficient screening of new treatments. Thanks to its short embryonic developmental time, small size, and its transparency, coupled with conservation of genetic mechanisms underlying biological processes and the possibility to transplant human cells; it represents an important model for gliomas studies (Figure 2D and Table 1). Compared to *Drosophila*, where modeling of brain tumor is achieved solely by production of transgenic models, zebrafish models of glioma are produced using different techniques.

#### Transgenic Models of Glioma

Gliomas can be induced in zebrafish by activation of the EGFR/RAS/ERK/AKT pathway *via* overexpression (a zic4 enhancer) of several oncogenes, such as *KRAS*^*V*12^, *EGFR^vIII^*, among others (Mayrhofer et al., 2017). Analysis of global RNA expression established that obtained brain tumors resemble GBMs of the mesenchymal subtype, with a strong YAP component. Similarly, overexpression of *KRAS*^*G*12*V*^ in a putative neural stem and/or progenitor cells induced brain tumorigenesis (Ju et al., 2015) and overexpression of dominant-active human Akt1 (*DAAkt1*) or Rac1G12V (*DARac1*) (Ptf1a promoter)-induced gliomas of various histological grades, frequently infiltrated (Jung et al., 2013). Different tumor-initiating cells affect the tumor type; in fact, overexpression of *KRAS*^*G*12*V*^ under the control of the krt5 gene promoter induced low frequency brain tumors in the ventricular zones (VZ) that resemble malignant peripheral nerve sheath tumors (MPNSTs) (Ju et al., 2015). In contrast, overexpression of *KRAS*^*G*12*V*^ under the control of the *gfap* gene promoter induced brain tumors characterized by prominent activation of the canonical RAS-RAF-ERK pathway in both VZs and brain parenchyma at higher frequency (Ju et al., 2015). This study demonstrated that zebrafish could be explored to study cellular origins and molecular mechanisms. Ju et al. (2014) also developed the first animal model of gliomagenesis driven by Sonic Hedgehog (Shh) by overexpressing Smoothened (Smoa1) under the krt5 neural promoter. Transgenic zebrafish models have also been used to study the macrophage infiltration and contribution to tumor growth. Two publications clarified that expression of the human AKT1 oncogene in neural cells leads to tumor formation and significant increase in the macrophage and microglia populations that showed tumor-promoting functions (Chia et al., 2018). In particular, this is due to infiltration of macrophages from the peripheral area into the brain mediated *via* Sdf1b-Cxcr4b signaling. Cancer cells exploit the mechanisms used to mediating neuro-microglial *via* P2ry12 activation to promote their own proliferation (Chia et al., 2019). Finally, several transgenic models expressing IDH1 mutations were used to evaluate their roles in tumor development, but none of them led to glioma development, suggesting that further mutations are required (Gao et al., 2018).

#### Knockout Models

Several strategies have been used to produce knock out models to study the involvement of endogenous genes in glioma formation. Shin et al. (2012) used targeting-induced local lesions in genomes (TILLING) strategy to generate several null alleles of *nf1a* and *nf1b*. Thanks to these transgenic strains - they demonstrated that loss of nf1 is involved in tumorigenesis; in fact, adult *nf1a*^+/−^; *nf1b*^–/–^; *tp53*^*e7/e7*^ animals show an earlier onset and increased penetrance of HGGs and MPNSTs (Shin et al., 2012). The same group also induced the CRISPR/Cas9-mediated knockout of *atrx*, a known tumor suppressor gene in sarcomas, in the *nf1/p53*-deficient zebrafish, to study its contribution to malignant growth (Oppel et al., 2019). Moreover, knock out models have also been used in combination with transgenesis to study the connection between the immune system and tumor growth, as described above (Chia et al., 2019).

#### Knockdown With Morpholinos

The MO are antisense oligonucleotides that bind to complementary target mRNAs and block their translation, acting similarly to small interfering RNAs (siRNAs) and short hairpin RNAs (shRNAs), or alter the pre-mRNA splicing. Gliomas and, in particular, glioblastomas are tumors characterized by a high level of vascularization, and their growth depends on the formation of new blood vessels (Ahir et al., 2020). MO have been used in zebrafish to study the role of genes in angiogenesis and their contribution to GBM growth. For example, this has been done with *Ephrin-B3* (*ephrinb3-like* in zebrafish), highly expressed in GBM and acting as a survival factor (Royet et al., 2017) and with *PlexA*, also highly expressed in GBM and in tumor-associated blood vessels in patient biopsies (Jacob et al., 2016).

#### Xenotransplantation of Cancer Cells

Xenotransplantation of human glioma cells in zebrafish is a powerful technique to study tumor growth and invasion. In fact, the transplanted cells not only survive but are also able to migrate and interact with the host environment. Zebrafish offers several advantages over other xenograft models: firstly, it lacks an adaptive immune system in early stages of life (before 30 days post-fertilization, pfs) (Lam et al., 2004); therefore, no use of immunocompromised animals is needed at these stages. Moreover, the presence of many genetic tools, together with the small size and the transparency of zebrafish, allows live and high-resolution imaging of the transplanted cells (Pudelko et al., 2018; Vargas-Patron et al., 2019). Xenotransplantation can be performed at different injection sites: glioma/glioblastoma cell lines as U87 and U373T have been injected in the yolk sack at the stage 48-h pfs and analyzed for several parameters such as survival, proliferation, and invasion (Yang et al., 2013a; Lai et al., 2017). These studies led to the characterization of the role of genes such as Rac, MMP-9 in glioma aggressiveness/invasiveness, or the role of TGF-β1 in enhancing tumor-induced angiogenesis (Yang et al., 2013b).

Injections have also been performed in the zebrafish brain, ranging between 48- and 72-h pfs. Two studies used modified U-87 glioma cells lines to characterize the role of the KMT2A-NOTCH regulatory cascade (Huang et al., 2017) and of RECQ1 Helicase (Vittori et al., 2017) in glioma proliferation. Other groups used xenografts injection in the brain to study microenvironment and tumor migration/invasion; Breznik et al. (2017) investigated the role of mesenchymal stem cells (MSCs) in modulation of glioblastoma cells invasion by xenotransplantation of a mixture of MSC and U87 and U373 GBM cells. Xenotransplant into the brain (7 days pfs) was used to study differentiation of primary GBM cells induced by treatment with Wnt ligands, or overexpression of β-catenin (Rampazzo et al., 2013). Recently, it has been reported an optically clear mutant zebrafish (*prkdc−/−, il2rga−/−*) that lacks adaptive and natural killer immune cells that have been used to graft GBM9 glioblastoma cells intraperitoneally at 2 months old, and seems to be a promising model for developing personalized therapeutic approaches (Yan et al., 2019).

In conclusion, zebrafish overcomes many drawbacks of murine models, in terms of tumor live visualization, late development of the immune response, and presence of many genetic tools for manipulation and of the *Drosophila* models, especially thanks to the higher degree of physiological similarity to the mammalian models. However, open questions remain on pharmacokinetic studies for accurate drug delivery, dosing, and metabolism in zebrafish due to the divergent physiological features (such as body temperature and organ systems) (Kirchberger et al., 2017; Casey and Stewart, 2020).

### 3D Organoid-Based Models for Glioma

Organoid models represent the most advanced approach, combining the most recent techniques of 3D culturing, 3D printing, and bioengineering (Rodrigues et al., 2021). There are two main cellular sources for deriving tumor organoids: the patient tumor biopsies (Figure 2E and Table 1) and pluripotent stem cells derived brain organoids (Figure 2F and Table 1).

#### Patient-Derived Organoids

The first glioblastoma organoids were generated by Hubert et al. in 2016. Contrary to the classical spheroids formed by one cell type, they created more complex organoids starting from xenografts, GEMMs, and patient biopsies (Hubert et al., 2016). They observed that such cells cultured using Matrigel-based 3D culture methods formed organoids, recapitulating many GBM features, including a hypoxic gradient and resistance to radiation. Recently, Jacob et al. (2020) have established a growth factors-free chemically defined medium-based protocol to derive, expand, and cryopreserve GBM organoids, starting from tumor biopsies, so called PDOs (patient-derived glioblastoma organoids). The cellular composition, as well as the transcriptional profiling, confirmed the high similarity between PDOs and the original tumor. Such similarities were also observed in the response to treatment with radiations and temozolomide, and the infiltrative nature was observed upon xenograft in nude mice. Organoids were also generated from glioma of different grades (Grades II, III, and IV) but, despite some of the original tumor features, are retained; they survive in culture for a limited time, making the biobanking and expansion quite challenging. Nevertheless, Golebiewska et al. (2020) were able to successfully generate a living collection of PDOs that maintains most of the genetic, molecular, and phenotypical features of the primary tumor and a similar response to therapy. They also analyzed the DNA methylation patterns, currently considered the gold standard for the correct brain tumor diagnosis and subtyping, which revealed a good correlation between primary tumor and the respective-derived PDO. Finally, PDOs have been shown to maintain some of the immune cells (i.e., microglia and T-cell) during the 1st weeks in culture, opening the possibility to study their interaction with tumor cells (Jacob et al., 2020). Nevertheless, culture conditions need to be improved to ensure a stable expansion of the immune compartment in long-term culture.

#### Genetically Engineered Brain Organoids

Few years ago, two independent studies showed how genetically modified brain cancer models can also be generated *in vitro*. Using the same *in vivo* approach of genes in human gliomas (i.e., *HRAS*^*G*12*V*^ and *TP53*^–/–^; *cMYC* overexpression; *EGFRvIII* and *EGFR* overexpression and *CDKN2A*^–/–^; *NF1*^–/–^ and *PTEN*^–/–^ and *TP53*^–/–^), cerebral organoids derived from human-induced pluripotent stem cells (hiPSCs) were modified in order to induce neoplastic organoids (Bian et al., 2018; Ogawa et al., 2018) (Figure 2F and Table 1). The addition of fluorescent reporter genes allows to specifically track the modified cells and as they grew. Such neoplastic organoids exhibited a transcriptional profile partially similar to human gliomas, and their aggressiveness was confirmed *in vivo* upon orthotopic injection into nude mice. However, they did not show strong molecular similarity to patients, and it was not clearly shown whether they also retain intra-tumoral heterogeneity. This represents the major weakness for making this model suitable to investigate the intra-tumoral heterogeneity. On the other hand, these models have a great potential in drug screening by providing “unlimited” mini tumor organoids. They can be genetically modified in a less time-consuming and more cost-effective way than mouse models, allowing for the screening of new genes involved in tumor formation before testing them *in vivo*. Finally, GEBOs might be a valid model to investigate the cell of origin of glioma as already shown to be feasible for Group 3 Medulloblastoma (Ballabio et al., 2021). Generation of cerebellar as well as cerebral organoids from hiPSCs relies on well-established protocols (Muguruma et al., 2015; Velasco et al., 2019) that generate structures resembling fetal cerebellum or brain also from molecular points of view (Velasco et al., 2019; Nayler et al., 2021).

#### GLICO

Cerebral organoids have been also recently used for studying the behavior and invasion of GBM cells. The first approach consisted of co-culturing GBM spheroids with mouse ESC-derived brain organoids, proving the potential of modeling GBM invasion with organoids (da Silva et al., 2018). Similarly, Linkous et al. (2019) described a model of co-culturing GBM-derived GSCs with brain organoids, called GLICO (Figure 2G). With this approach, they were able to detect GBM cells invading normal brain organoids using microtubule structure, similar to what is observed in the GBM cells invading human brain parenchyma (Osswald et al., 2015). Additional studies have shown that this model can be successfully used to investigate invasion (Goranci-Buzhala et al., 2020; Krieger et al., 2020). Furthermore, when GSCs were co-cultured in 3D with organoids starting to differentiate, allowing also to recapitulate the lineage heterogeneity of cancer cells (Pine et al., 2020; Azzarelli et al., 2021). Nevertheless, this phenomenon seemed to be also dependent on the organoid media composition (Azzarelli et al., 2021). The pitfalls of GLICO are those related to being based on cell lines that do not allow investigating cancer in a context where original cell types and microenvironment are maintained (Jacob et al., 2020). Nevertheless, GLICO represents an excellent and valid model for studying cell behavior, such as invasion and cellular plasticity of GSCs.

#### Bioprinted Glioblastoma Organoids

The concept of bioprinting GBM organoids consists of putting together different cell types to recreate the different tumor regions. For example, it is possible to print freshly dissociated GBM cells on a chip together with vascular endothelial cells using a porcine ECM as “bioink” (Yi et al., 2019). This GBM-on-a-chip approach also allows recreating the hypoxic gradient and the infiltrating region. To increase complexity, GBM stem cells have been bioprinted together with neural stem cells (NSCs), astrocytes, and macrophage. The presence of immune cells allows to study their interaction and effect on the growth and invasion of cancer cells (Tang et al., 2020). Despite these models are very promising, the approach is still in its infancy and, therefore, presents some pitfalls, such as the technology is not yet precise and requires the use of specialized equipment and skilled researchers that, in turn, require significant investments.

To summarize, as for the other models, organoid-based systems have advantages and disadvantages. As an *in vitro* model, the usage of organoids allows to perform a drug screen and a test for immunotherapy; in addition, organoids can be used for an invasion assay and for testing the function of genes for inducing tumors. On the other hand, such models have disadvantages such as lack of vascular network. In the future, the possibility to induce cancer in “vascularized” brain cancer organoids might overcome this limitation and being used to study *in vitro* the interaction between cancer cells and blood vessels (Shi et al., 2020; Matsui et al., 2021). Concerning the intra-tumoral heterogeneity, this can be observed only when organoids are generated from tumor biopsies (Jacob et al., 2020). Finally, another challenge in the field remains to create brain cancer organoid with proper tumor microenvironment such as an immune compartment. Indeed, it would be worth establishing co-culture of brain cancer organoids (i.e., PDO) with different types of immune cells derived from patient peripheral blood or tumor biopsies (i.e., tumor infiltrating lymphocytes or tumor-associated macrophages/microglia). Alternatively, following a hiPSC-based approach, GEBOs might be co-cultured with iPSC-derived microglia (Xu et al., 2021).

## Medulloblastoma

Medulloblastoma (MB) is the most common malignant brain tumor in childhood that specifically arises in the cerebellum. The highest peak of incidence is during the first decades of life, even though this disease can occur throughout adulthood (Louis et al., 2016). Nowadays, an effective and definitive treatment has not yet been found, and 40% of affected children experience tumor recurrence, while 30% die from MB (Jones et al., 2012). Next-generation techniques have been applied to analyze MB molecular features/profiles. Genome-sequencing and array-based transcriptional profiling allowed the classification of MB into four main molecular subgroups: WNT, SHH, Group 3, and Group 4 (Taylor et al., 2012). The classification depends on a broad variety of macro and micro-genetic aberrations, which define specific cytogenetics, mutational patterns, gene expression signatures, and patient outcomes. Because of its molecular subtyping, here, we give an overview of the different models developed for each MB subgroup.

### Mouse Models of Medulloblastoma

#### WNT Medulloblastoma

The gold GEMM of WNT MB comes from the efforts of Gibson et al. (2010), hypothesizing, for the first time, the cell of origin of the tumor. Human WNT MB are distributed within the IV ventricle and infiltrate the brainstem. Indeed, genes marking human WNT MB are more frequently expressed in the lower rhombic lip (LRL) and embryonic dorsal brainstem. For this reason, Gibson and collaborators generated *Blbp-Cre*^+^; *Ctnnb1*^+/lox(ex3)^; *Tp53*^flx/flx^ mice, characterized by a dominant mutation of the beta-catenin gene and loss of the tumor suppressor *Tp53* in *Blbp*^+^ cells, which are Olig3^+^ progenitor cells in the LRL and progenitor cell populations across the hindbrain (including the cerebellar ventricular zone) (Gibson et al., 2010) (Figure 2B and Table 1). *Blbp-Cre*^+^; *Ctnnb1*^+/lox(ex3)^; *Tp53^flx/flx^* mice developed MB, recapitulating the anatomy and gene expression profiles of human WNT MB, as well as the aberrant vasculature that interferes in the blood brain barrier formation. A damaged blood brain barrier could increase the drug’s efficacy, thus explaining the good prognosis and excellent response to chemotherapy compared to the other MB subgroups. These data indicate the similarities between the GEMMs and the human patients, showing their importance in defining new therapeutic approaches (Phoenix et al., 2016). A tumor suppressor role has been recently hypothesized for *DDX3X* (Patmore et al., 2020), often found mutated in WNT MB. DDX3X regulates hindbrain patterning, and its loss removes lineage restriction toward tumor formation, allowing for the onset of WNT MB from either lower or upper rhombic lips.

#### SHH Medulloblastoma

The first GEMM for SHH MB was a *Ptch1*^+/−^ model, where aberrant SHH pathway activation leads to uncontrolled proliferation of cerebellar granule neuron progenitors (CGNPs) (Goodrich et al., 1997). Indeed, granule lineage identity is a prerequisite for the SHH MB onset, as it has been demonstrated by the conditional *Ptch1* knockout in unipotent (*Math1*^+^) CGNPs (Schüller et al., 2008; Yang et al., 2008) and through the deregulation of different effectors of the SHH pathway, such as Smoothened and Sufu (Lee et al., 2007; Dey et al., 2012) (Figure 2B and Table 1). Based on mutations found in human SHH MB, a plethora of GEMMs have been generated using different approaches (Wetmore et al., 2001; Uziel et al., 2005) (listed in Table 1). A subgroup of patients with SHH MB shows somatic loss-of-function mutations of the transcriptional corepressor *BCOR*. Interestingly, *BCOR* is involved in MB formation (Tiberi et al., 2014; Kutscher et al., 2020) with possible therapeutic implications in *BCOR* mutant SHH MB. Zuckermann et al. (2015) have shown an interesting method to validate the oncogenic role of mutations found in human patients by performing postnatal somatic CRISPR/Cas-mediated deletion of tumor suppressor genes using a polyethylenimines-mediated *in vivo* transfection into the mouse neonatal cerebellum. Indeed, *Ptch1* CRISPR/Cas-mediated deletion in mice characterized by homozygous deletion of *Trp53* recapitulates MB (Zuckermann et al., 2015). SHH MB can be obtained by different cell types, other than CGNPs, such as cochlear granule neuron progenitors (CNPs) (Grammel et al., 2012). Recently, we have proposed postmitotic granule neurons as a possible origin for the human adult SHH MB. These cells can be reprogramed and give rise to tumors in the cerebellum of mice resembling human adult SHH MB (Aiello et al., 2019). In particular, the co-expression of mutant *Brfp1* and *SmoM2* alters chromatin accessibility of stem/progenitor-related genes, thus reprogramming the precursor cell properties and favoring the adult SHH MB tumorigenesis. However, whether the de-differentiation process of granule neurons is a key event in *BRPF1*-mutated SHH MB is still unknown and needs further investigation. Additionally, SHH GEMMs were brought to the discoveries of new important pathological features. Indeed, single-cell RNA sequencing on murine Shh MB treated with vismodegib (a Smo inhibitor) revealed some cell types (*Sox2*^+^ and *Myod1*^+^) resistant to treatment (Ocasio et al., 2019). A rare Sox2^+^ quiescent cell population, enriched after antimitotic chemotherapy, and Smoothened inhibition are thought to be responsible for tumor relapse (Vanner et al., 2014). Furthermore, the tumor sustains itself by shaping its surroundings to make it conducive to growth (Yao et al., 2020). CGNPs are, indeed, able to *trans*-differentiate into tumor astrocytes that sustain tumor progression by activating microglia *via* IL-4 production. Microglia, in turn, produce IGF-1 that promotes tumor progression. Among the mechanisms driving tumor resistance to conventional therapies and relapse, it has been shown the presence of a stem cell niche within SHH MB, namely cancer stem cells (CSCs), which express the stemness marker Nanog under Hh/Gli transcriptional regulation (Po et al., 2010; Miele et al., 2017; Abballe and Miele, 2021).

#### Group 3 Medulloblastoma

Modeling of Group 3 MB is more challenging due to the intratumoral heterogeneity and the similarities to Group 4 in the mutational profile. For years, the GTML mouse model has been considered as the golden standard model for Group 3 MB. This transgenic mouse is characterized by a Tet system that allows the expression of both *MYCN* and *Luciferase* under the control of the Glutamate transporter 1 (Glt1) promoter, reported to be expressed in hindbrain progenitors (Swartling et al., 2010). Since several high-risk Group 3 human patients showing relapse after treatment are characterized by *MYC* amplification and *TP53* inactivating mutation, this model was exploited to verify the interaction between *P53* and MYCN. *GTML/Trp53*^KI/KI^** mice produce a more aggressive tumor that recapitulates the human large cell/anaplastic (LCA) histology and relapse (Hill et al., 2015). However, GTML-derived MB neurospheres show, in a small percentage, SHH-dependent features, thus not fully recapitulating the Group 3 MB human scenario (Swartling et al., 2012).

Due to the high diversity in the mutational landscape of Group 3 MB, we developed an *in vivo* screen approach to test putative tumor driver mutations among hits identified by exome sequencing and microarray data of human Group 3 patients with MB.

Either overexpressing oncogenes with the PiggyBac transposon system or by CRISPR/Cas9-mediated deletion of tumor suppressor genes, we found that *Gfi1* + *c-MYC* and *Otx2* + *c-MYC* genes overexpressions were able to mimic the histological and transcriptional profile of human Group 3 MB (Ballabio et al., 2020) (Figure 2B and Table 1). Moreover, the *Otx2* + *c-MYC* combination of genes generated metastasis, recapitulating the malignancy of the tumor. Chromatin modifiers such as *MLL4* (alias for *KMT2D*) are often mutated in Group 3 patients with MB, as already mentioned, and whether these could have a causative role in tumor formation has been tested. *Nestin-cre; Mll4*^flox/flox^** mice lead to the downregulation of several tumor suppressors, such as *Dnmt3* and *Bcl6*, and to Group 3 MB formation (Dhar et al., 2018). Despite the extensive genomic characterization, it is still unknown what the developmental origins of Group 3 MB are. We have hypothesized a critical role of Notch1 in tumor formation, claiming that its expression level in different progenitor cells impairs their competence in inducing MB. In particular, *S100b*^+^ cells show a higher level of Notch1 compared to *Math1*^+^ progenitor cells, and are able to initiate Group 3 MB upon *Gfi1* + *c-MYC* overexpression, while *Math1*^+^ and *Sox2*^+^ cells do not initiate tumorigenesis (Ballabio et al., 2021). The overexpression of *MYC* in isolated *TP53^–/–^* CGNPs and their following orthotopic transplantation in nude mice is able to mimic some of the Group 3 MB clinical features, strongly suggesting their cooperation in determining a more aggressive behavior (Kawauchi et al., 2012; Pei et al., 2012). However, Group 3 MB features can be also obtained by passing the *TP53* loss that is not often found within Group 3 tumors. Indeed, it has been shown that the overexpression of *c-Myc*, together with *GFI1*/*GFI1B* activation, allows to recapitulate Group 3 MB tumorigenesis (Northcott et al., 2014; Vo et al., 2017). A list of G3 GEMMs is presented in Table 1.

#### Group 4 Medulloblastoma

The Group 4 MB is the least explored and understood among the subgroups. A comparative analysis of protein phosphorylation levels between Group 3 and Group 4 leads to hypothesize a role of the receptor tyrosine kinase (RTK) pathway in the Group 4 MB onset; these findings were validated by the enrichment at the mRNA level of *ERBB4*, a well-established RTK member (Forget et al., 2018). SRC is a key regulator of the RTK pathway and was found upregulated at both mRNA and protein levels in Group 4. Moreover, *in utero* electroporation in the fourth ventricle of E13.5 mouse embryos of the active form of SRC, together with a dominant negative form of TP53, leads to Group 4 MB formation (Figure 2B and Table 1). However, *TP53* seems not to be mutated in Group 4 MB, but iso-chromosome 17q is frequently present in these patients. SRC-driven tumors present distinct molecular features from MYC-derived tumors. Since ERBB4 and SRC were detectable in the nuclear transitory zone (NTZ) on embryonic Day 13.5 (E13.5), Forget et al. (2018) speculated that their expression could reflect Group 4 developmental origin. ScRNAseq analysis on human Group 4 MB indicated an enrichment of markers involved in neuronal differentiation, such as *ENO2*, *SYT11*, *TUBB3*, and *MAP2*, or in glutamatergic lineage specification, such as *EOMES* and *LMX1A*. These factors have been implicated in defining neuronal fates of unipolar brush cells (UBC) and glutamatergic cerebellar nuclei (GluCN) in the embryonic upper rhombic lip (Englund et al., 2006; Chizhikov et al., 2010; Hovestadt et al., 2019). Lin et al. (2016) found enrichment of super-enhancer activation of LMX1A, TBR2, and LHX2, which could support origin from progenitors in the embryonic upper rhombic lip. However, the developmental origin of Group 4 is still an open question, and further studies must be conducted to clarify the involvement of UBC, GluCN, or other cell types in the Group 4 MB onset. The identification of the cell of origin is a crucial step for the development of faithful and proper models for Group 4 MB.

### Patient-Derived Xenograft and Patient-Derived Organoids Xenograft

As previously stated, mouse models show some limitations due to the different biological contexts and cannot fully recapitulate the complexity of human tumors (Gould et al., 2015). For this reason, PDX models emerged as an important tool to investigate subtype specific features of different pediatric brain tumors. A study conducted by the Children’s Oncology Group set the generation of 30 orthotopic pediatric brain tumor PDX models (Brabetz et al., 2018). An important effort in generating PDX models has been also achieved by the St. Jude Children’s Research Hospital by the generation of 37 novel orthotopic PDX models derived from patients with pediatric brain tumor (Smith et al., 2020). These new models include all the four MB subgroups (WNT, SHH, Group 3, and Group 4) that have been shown to maintain histological features of the original tumors and to genetically match the patients’ tumors. DNA methylation, transcriptional and histological analyses at early and late passages demonstrated the reliability of PDX models, providing a useful and valid resource to study cancer biology and to test novel and tailored therapeutic approaches.

Recently, it has been generated a humanized mouse model based on the orthotopic transplantation of human neuroepithelial stem cells (NES) (Huang et al., 2019). NES are multipotent stem cells able to differentiate into neurons with hindbrain identity. Orthotopic transplantation of NES transduced with *MYCN* leads to the formation of a human cancer phenotype as a powerful tool to dissect the processes of tumorigenesis. An alternative method to model SHH MB using human cells was described by Susanto et al. (2020) that performed an orthotopic transplantation of iPSC-derived NES from a Gorlin patient with a germline *PTCH1* mutation. They followed tumor development by re-transplanting tumor-isolated NES (tNES) cells in nude mice and identifying *LGALS1* as a putative new therapeutic target.

### 3D Organoid-Based Models for Medulloblastoma

Similar to gliomas, 3D cerebellar organoid models have been shown to be an emerging important tool to study human medulloblastoma. Taking advantage of already-available exome sequencing data and after adapting a previously developed protocol for cerebellar organoid generation (Muguruma et al., 2015; Ballabio et al., 2020), the first organoid model of Group 3 MB has been generated by overexpressing a combination of *Gfi1* + *c-MYC* and *Otx2* + *c-MYC onco*genes, identified previously as candidate driver genes in the same study by an *in vivo* mutagenesis screen. These Group 3 MB organoid models more closely mimic the histological, transcriptional, and DNA methylation profile of human Group 3 MB (Ballabio et al., 2020, 2021) (Figure 2B and Table 1), suggesting that they could be a more suitable platform for high-throughput drug testing and development of personalized therapies (Qian et al., 2019). Future studies could be done to develop models for the other MB subtypes by mutagenesis or by engraftment of patient-derived tumor cells. These could then allow us to study with more precision the mechanisms at the origin of the tumor formation and how the mutations found in the patients’ tumors drive tumorigenesis with unprecedented details in a human tissue. This could then lead to the identification of new potential targets for therapies and for diseases that, to date, cannot be tackled. It is, indeed, also possible that the human brain, due to its species-specific differences, could have different susceptibility to disease and brain tumors (Watanabe et al., 2017; Kanton et al., 2019; Eichmüller et al., 2022), a reason why studying mechanisms of tumorigenesis in human tissue becomes essential.

## Ependymoma

Ependymoma can originate in the brain or spinal cord. In the brain, ependymomas are thought to originate from ependymal cells lining in the ventricles. Histologically, ependymomas are classified into four groups: subependymoma (WHO Grade I), myxopapillary ependymoma and classic ependymoma (WHO Grade II), and anaplastic ependymoma (WHO Grade III), of which classic and anaplastic ependymoma are the most common subtypes in children (Figure 1). Over 90% of pediatric ependymomas arise in the infratentorial and supratentorial regions. Supratentorial (ST) ependymomas in children have two major subgroups: *RELA* fusion-positive ependymoma and *YAP1* fusion-positive ependymoma. Ependymoma mouse models have been generated by *RELAFUS1* fusion gene expression in *Nestin*^+^, *Gfap*^+^ or *Blbp*^+^ cells in the mouse brain. These tumors recapitulate some of the histology and the transcriptome panel of human ependymomas (Ozawa et al., 2018). The *YAP1-MAMLD1* fusion gene delivered to mice by *in utero* electroporation drives tumor formation, and tumors share histological and molecular characteristics of human ependymoma (Pajtler et al., 2019). Recently, Eder et al. (2020) have reported that ectopic expression of active nuclear YAP1 (nlsYAP5SA) or conditional deletion of YAP1’s negative regulators *Lats1* and *Lats2* kinases in neural progenitor cells of the ventricular zone also induced tumors with molecular characteristics of human ependymoma. PDXs derived from pediatric ependymoma showed to maintain a genetic, molecular, and histological similarity to the parental tumors, opening the possibility to have an additional model for investigating and treating such a disease (Brabetz et al., 2018; Smith et al., 2020). No organoid-based models have been reported for human ependymoma (Table 1).

## Meningiomas

Meningioma is another common type of tumors located in the brain, which originates from the meninges. Meningiomas, representing the most frequent tumor in the adult, are generally benign (WHO Grade I) with a lower percentage classified as atypical (WHO Grade II) and rarely anaplastic (WHO Grade III) (Boetto et al., 2021). The genomic profile of a large cohort of meningiomas has identified alteration in the genes encoding for NF2, SMARCB1, SMARCE1, TRAF7, KLF4, POLR2A, BAP1, and members of the PI3K and Hedgehog pathways (Youngblood et al., 2019). The mouse model of meningiomas consisted in xenograft of immortalized cell lines or patient-derived tumor cells (McCutcheon et al., 2000; Püttmann et al., 2005; Cargioli et al., 2007; Ragel et al., 2008), GEMMs (Kalamarides et al., 2002, 2011; Peyre et al., 2013, 2015; Boetto et al., 2018, 2021) and syngeneic allogenic graft (Boetto et al., 2021) (Table 1). Meningiomas can be induced in GEMMs overexpressing *PDGFB* in a context of loss of function of *Nf2*, *Cdkn2ab* or *p16Ink4a* or over-expressing only *SmoM2* PGDS^+^ arachnoid cells. Recently, PDO has been generated also from meningioma biopsies (Chan et al., 2021; Yamazaki et al., 2021). Its histological and molecular characterizations positively confirmed the high similarity between the parental tumor and PDOs (Yamazaki et al., 2021).

## Translation in Clinical Practice of Model-Based Knowledge and Discoveries

The main goal of the different models is to be used for the design of new therapeutical approaches and then being translated into clinical practice for glioma treatments. Concerning high-grade glioma (including GBM), the standard chemotherapeutic treatment is based on temozolomide, while lower grade gliomas relies on procarbazine, lomustine, and vincristine or temozolomide. Despite the enormous number of pre-clinical *in vivo* and *in vitro* models, the drugs used for treating gliomas (from Grade II to Grade IV) have remained the same over the last decades. Indeed, this negative and low progression of developing new drugs against glioma has also pushed the field to test already approved drugs originally designed for other diseases (Lyne and Yamini, 2021). This approach, called drug repositioning, has the advantage to have drugs already being tested for their safety, making eventually the path to a clinical trial to be faster. Furthermore, this approach could be coupled to the possibility to generate, expand, and Biobank organoids derived from histologically and molecularly different tumors (Golebiewska et al., 2020; Jacob et al., 2020) in order to have a more “personalized” and specific drug screen.

Current treatment for MB consists of surgery, radio- and chemotherapy; nevertheless, after surgery, patients suffer from severe psychosocial, neurocognitive, and neuro-endocrine deficits. Present studies are focused on the development of target therapies that take into account the molecular differences among the subgroups (Northcott et al., 2019; Thompson et al., 2020). In the scenario, the development of *in vitro* and *in vivo* models is crucial. In general, despite the models developed for brain cancer are increasing in terms of numbers, complexity, and the degree of similarity to the original tumor, the drugs available for treating the disease are always the same. What has really improved tough is the knowledge of the genetics and biology of the different types of brain cancer. This could be considered the starting point for rethinking about new pharmacological, immunological, and genetic therapies.

## Conclusion

Genomic, transcriptomic, and proteomic analyses on human brain tumor patients have greatly increased our knowledge and understanding of the signaling pathways regulating the different cancer subtypes. Through the exploitation of these data, several models have been created to mimic the pathogenesis and to gain knowledge about the molecular mechanisms of the tumors. All the brain tumor models developed so far have shown some pitfalls in the correct modeling of the disease and the human translation of the findings (Figure 2). The first issue is related to the genetics, with all the mouse models and hiPSCs derived-brain organoid, relying only on a few of the genetic abnormalities found in patients with brain tumor. In this direction, it will be worth expanding the combination of genetic abnormalities inducing tumor in mice and organoids performing large genetic screens. Drosophila represents a valid complementary model to be used for performing a “low-cost” initial genetic screen for a new putative cancer driver. Secondly, the drug screen platforms used so far have mostly relied on 2D-cultured tumor cell lines. Therefore, the use of hiPSCs-derived brain organoid represents a unique platform for medium/high throughput screening of new molecules to hinder tumor progression. On the other hand, organoid cultures have some limitations. The lack of vascularization leads to reduced oxygenation and access to nutrients, with the consequent formation of an inner necrotic core in the tissue when cultures are kept for long-term studies. The absence of immune cells and microglia makes the studies of tumor-microenvironment preferable *in vivo*. Some limitations can be overcome at the current state-of-the-art by transplanting human organoids in the mouse brain (Mansour et al., 2018; Ballabio et al., 2020; Bhaduri et al., 2020). Furthermore, the ability to cultivate the original human primary tumor with PDO will be a breakthrough that will provide the possibility to personalize the treatments. Recently, patient-derived xenografts (PDXs) have been shown to be the most advanced model, recapitulating the human disease. These models might be used to model frequent as well as rare childhood brain tumor entities, including HGG, MB, anaplastic ependymoma, atypical teratoid rhabdoid tumor, and embryonal tumor (Brabetz et al., 2018; Smith et al., 2020). PDOX models will be valuable platforms for evaluating novel therapies and conducting pre-clinical trials to accelerate progress in the treatment of pediatric brain tumors. However, the usage of immunocompromised mice does not allow to investigate the interaction between tumor cells and immune system. Moreover, since PDO and PDX are derived from tumor biopsies, they should not be considered suitable for investigating the neoplastic transformation of brain cells. In this review, we depicted an overview of the *in vitro* and *in vivo* brain tumor models used in the past together with the most technologically advanced ones. Considering the entire pros and cons described in this review, the best model system must be chosen, depending on the specific biological question that needs to be addressed.

## Author Contributions

FA, GA, AS, and LT made substantial contributions to researching data for the article, to discussions of content, and to writing the manuscript. EM and LA reviewed and edited the manuscript before submission. All authors contributed to the article and approved the submitted version.

## Conflict of Interest

The authors declare that the research was conducted in the absence of any commercial or financial relationships that could be construed as a potential conflict of interest.

## Publisher’s Note

All claims expressed in this article are solely those of the authors and do not necessarily represent those of their affiliated organizations, or those of the publisher, the editors and the reviewers. Any product that may be evaluated in this article, or claim that may be made by its manufacturer, is not guaranteed or endorsed by the publisher.
